# Sex-related presentation, outcome, and treatment in CICU: the SPOTlight STUDY

**DOI:** 10.1007/s00392-025-02816-z

**Published:** 2025-12-08

**Authors:** Alessia d’Aiello, Mattia Brecciaroli, Simone Filomia, Daniela Pedicino, Marco Giuseppe Del Buono, Gianluigi Saponara, Rocco Antonio Montone, Gaetano Pinnacchio, Lorenzo Genuardi, Laura Antenucci, Jacopo Lenkowicz, Giovanna Liuzzo, Carlo Trani, Francesco Burzotta, Tommaso Sanna

**Affiliations:** 1 Department of Cardiovascular and Pulmonary Sciences, Catholic University of the Sacred Heart, Rome, Italy; 2 Department of Cardiovascular Sciences, Fondazione Policlinico Universitario A. Gemelli IRCCS, Rome, Italy; 3 Fondazione Policlinico Universitario A. Gemelli IRCCS, Rome, Italy

**Keywords:** Sex differences, Cardiac Intensive Care Unit (CICU), Cardiogenic shock, Gender-specific health strategies

## Abstract

**What is already known:**

• Sex-related differences in cardiovascular disease presentation, management, and outcomes have been increasingly recognized in recent years.

• Women with acute cardiovascular conditions often receive less aggressive diagnostic and therapeutic interventions compared to men, particularly in the setting of acute coronary syndromes.

**What this study adds:**

• This study provides a comprehensive evaluation of sex-related differences in a contemporary cohort of patients admitted to the Cardiac Intensive Care Unit (CICU), highlighting distinct admission patterns and management disparities.

• Women with cardiovascular disease tend to be older at presentation, exhibit different risk factor profiles, and show distinct clinical manifestations compared to men.

• It identifies higher mortality rates for women with STEMI and pulmonary embolism, emphasizing the need for improved recognition and tailored management of sex-specific differences in acute cardiovascular care.

**Graphical Abstract:**

**Supplementary Information:**

The online version contains supplementary material available at https://doi.org/10.1007/s00392-025-02816-z.

## Abstract (highlights)

**Background:**

Sex-related differences in cardiovascular medicine have gained increasing attention recently. This study aims to provide an extensive evaluation of sex-related differences in acute cardiovascular care, focusing on admission patterns, management strategies, and outcomes of patients admitted to the Cardiac Intensive Care Unit (CICU).

**Methods:**

This retrospective, single-center observational study analyzed CICU admissions at Fondazione Policlinico Gemelli IRCCS from November 2020 to April 2024. We analyzed the frequency of cardiovascular conditions, diagnostic and therapeutic intervention utilization, and in-CICU mortality in the female population. We investigated CICU length of stay (LOS) and survival at follow-up.

**Results:**

Among 2,541 patients admitted to the CICU, 33% were women. Women were significantly older than men and had a different cardiovascular risk profile, with lower rates of smoking and diabetes but a higher prevalence of obesity. The most common reasons for CICU admission in women were acute heart failure, acute presentations of valvular heart disease, acute coronary syndromes and atrioventricular block (AVB). Women had a higher incidence of AVB, Takotsubo syndrome, and pericardial diseases but a lower incidence of acute coronary syndromes. Women with STEMI were less likely to undergo coronary angiography, PCI, and CABG. Overall, LOS and in-CICU mortality were similar between the sexes, but women had higher mortality rates for STEMI and pulmonary embolism. In the long term, women showed significantly higher mortality.

**Conclusions:**

Our study highlights significant sex-specific differences in patients admitted to the CICU and the need for improved recognition of sex-specific differences in cardiovascular disease, ensuring equitable access to timely and appropriate interventions.

**Supplementary Information:**

The online version contains supplementary material available at https://doi.org/10.1007/s00392-025-02816-z.

## Graphical abstract

**Figure:**
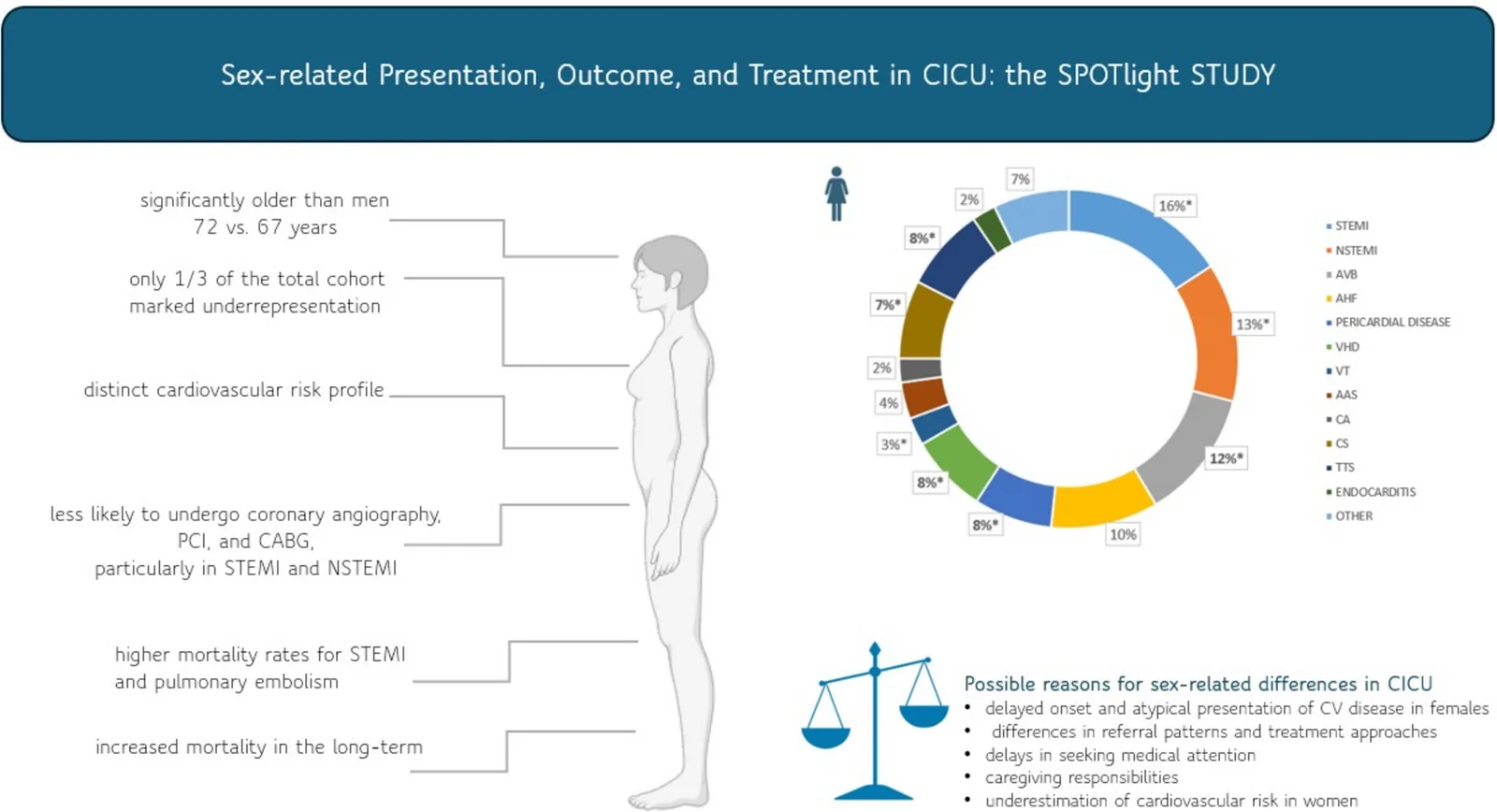

## Background

Sex-related differences in cardiovascular medicine have gained increasing attention in recent years, highlighting significant variations in disease prevalence, symptomatology, severity, therapeutic response, and prognosis between men and women [1]. However, most existing research focuses on individual conditions or specific aspects of cardiovascular management, rather than offering a comprehensive evaluation of sex differences in cardiovascular care needs. This knowledge gap is particularly pronounced in acute cardiovascular care. A deeper understanding of sex-related disparities is crucial for improving risk stratification, resource allocation, and personalized treatment strategies in the acute setting. This study aims to fill this gap by providing a comprehensive evaluation of sex-related differences in acute cardiovascular care, focusing on admission patterns, management strategies, and both short- and long-term outcomes of patients admitted to the CICU.

## Study objectives

The primary objective of this study is to explore the reasons for hospitalization in Cardiac Intensive Care Unit (CICU) among female patients.

The secondary objective of this study is to evaluate the clinical course of female patients during their CICU stay by assessing the diagnostic and therapeutic procedures performed, length of stay, mortality rates, and survival outcomes after discharge.

## Methods

### Study population and setting

We conducted a retrospective, single-center observational study, enrolling 2,541 consecutive adult patients admitted to the Cardiac Intensive Care Unit at Fondazione Policlinico Universitario Agostino Gemelli IRCCS in Rome, Italy, between November 1, 2020, and April 30, 2024. The general admission criteria, detailed in Supplementary Material 1, are consistent with established recommendations in the literature. A STROBE-compliant flow diagram illustrating the selection of the study population is provided in Supplementary Material 2.

The Fondazione Policlinico Universitario Agostino Gemelli IRCCS is a surgical/interventional tertiary Hub centre. Our center is structured to provide intensive care across multiple specialized units, including a general intensive care unit, cardiac and cardiac surgery intensive care unit, and neurocritical, postoperative, pediatric, and neonatal intensive care units. The hospital does not operate an active heart transplant or left ventricular assist device (LVAD) program.

### Data collection

Data on ICD-9 CM diagnoses, length of hospitalization, procedures performed, in-hospital outcomes and follow-up survival data have been retrieved through the Gemelli Generator Real World Data (G2 RWD) facility. Key steps included automated anonymized data extraction, quality checks, data transformation, and integration (Supplementary Material 3). In this study, the primary diagnosis was defined as the condition that directly prompted the patient’s admission to the cardiac intensive care unit. Any other coexisting diagnoses present at the time of admission were classified as secondary, as they were not the principal determinants for CICU. The study has been approved by the Ethics Committee of Lazio Area 3 (ID 7293).

### Statistical analysis

Considering the exploratory nature of the primary endpoint and the retrospective design of our study, no formal sample size calculation was performed. Continuous variables were expressed as means ± standard deviations, and categorical variables were presented as frequencies and percentages. Comparisons between groups were conducted using the independent samples t-test for continuous variables and the chi-square test (or Fisher’s exact test when appropriate) for categorical variables. For the comparison of clinical outcomes and procedures, odds ratios (ORs) with 95% confidence intervals (CIs) were calculated to assess the strength of associations between age groups and specific conditions or interventions. Statistical significance was set at a *p*-value of < 0.05. Survival time, expressed in days, was evaluated using the Kaplan–Meier method, accounting for censored data. Survival distributions between age groups and diagnoses were compared using the Log-rank test. All analyses were two-tailed, and a *p*-value of less than 0.05 was considered statistically significant. Statistical analyses were performed using IBM SPSS Statistics version 29.0 (IBM Corp., Armonk, NY, USA).

## Results

During the study period, 2,541 patients were admitted to our CICU. Among these patients, 831 (33%) were women, and 1710 (67%) were men. Women were significantly older than men, with a mean age of 72 vs. 67 years (*p* < 0.001), as detailed in Table 1. Significant differences in traditional cardiovascular risk factors were identified between female and male patients. Women were less likely than men to be active smokers (19.6% vs. 28%, *p* < 0.001) and former smokers (19.2% vs. 34.2%, *p* < 0.001). Additionally, the prevalence of diabetes mellitus (DM) was lower in women compared to men (24.1% vs. 30.5%, *p* < 0.001). In contrast, obesity (defined as BMI > 30 kg/m^2^) was significantly more frequent among women than men (11.1% vs. 8.5%, *p* = 0.049). No significant sex-based differences were observed for family history of heart disease (6% vs. 6.9%, *p* = 0.41), dyslipidemia (50.4% vs. 51.2%, *p* = 0.73), or hypertension (74.9% vs. 74.4%, *p* = 0.803). These findings are summarized in Table 2.

**Table 1:** Age distribution for sex

Sex	N_tot_	Age (years, mean)	Sd	*P*-Value	SMD
Male	1710	67.75	14.56	** < 0.001**	0.33
Female	831	72.64	14.65

**Table 2:** Differences in traditional cardiovascular risk factors between sexes

Traditional Cardiovascular Risk Factors	Male (n, %)	Female (n, %)	*P*-value	SMD
Active smoker	429 (28)	143 (19,6)	** < 0.001**	0.19
Diabetes	466 (30.5)	176 (24.1)	** < 0.001**	0.14
Family History of Heart Disease	106 (6.9)	44 (6)	0.41	0.03
Dyslipidemia	781 (51.2)	368 (50.4)	0.73	0.01
Hypertension	1136 (74.4)	547 (74.9)	0.803	0.01
Obesity (BMI > 30 kg/m^2^)	130 (8.5)	81 (11.1)	**0.049**	0.08
Former smoker	522 (34.2)	140 (19.2)	** < 0.001**	0.34

### Acute healthcare needs

The most frequently observed acute cardiac conditions requiring a CICU hospitalization in women were acute heart failure (including both de novo presentations and acute decompensations of previously known heart failure; AHF, 44.9%), acute presentations of valvular heart disease (AVHD, 22%), atrial fibrillation (AF, 17.8%), ST-elevation myocardial infarction (STEMI, 16.1%), non-ST-elevation myocardial infarction (NSTEMI, 15.3%), and high grade or 3rd degree atrioventricular block (AVB, 14.7%).

Compared to men, women were more likely to be diagnosed with AVB (14.7% vs. 9%, *p* < 0.0001), Takotsubo syndrome (TTS, 8.1% vs. 0.6%, *p* < 0.0001), pericardial effusion or tamponade (8.7% vs. 4.7%, *p* < 0.0001), pulmonary embolism (PE, 3.9% vs. 1.6%, *p* < 0.0001), cardiogenic shock (CS, 7.5% vs. 5.4%, *p* = 0.042), and AVHD (22% vs. 12.9%, *p* < 0.0001).

**Fig. 1:**
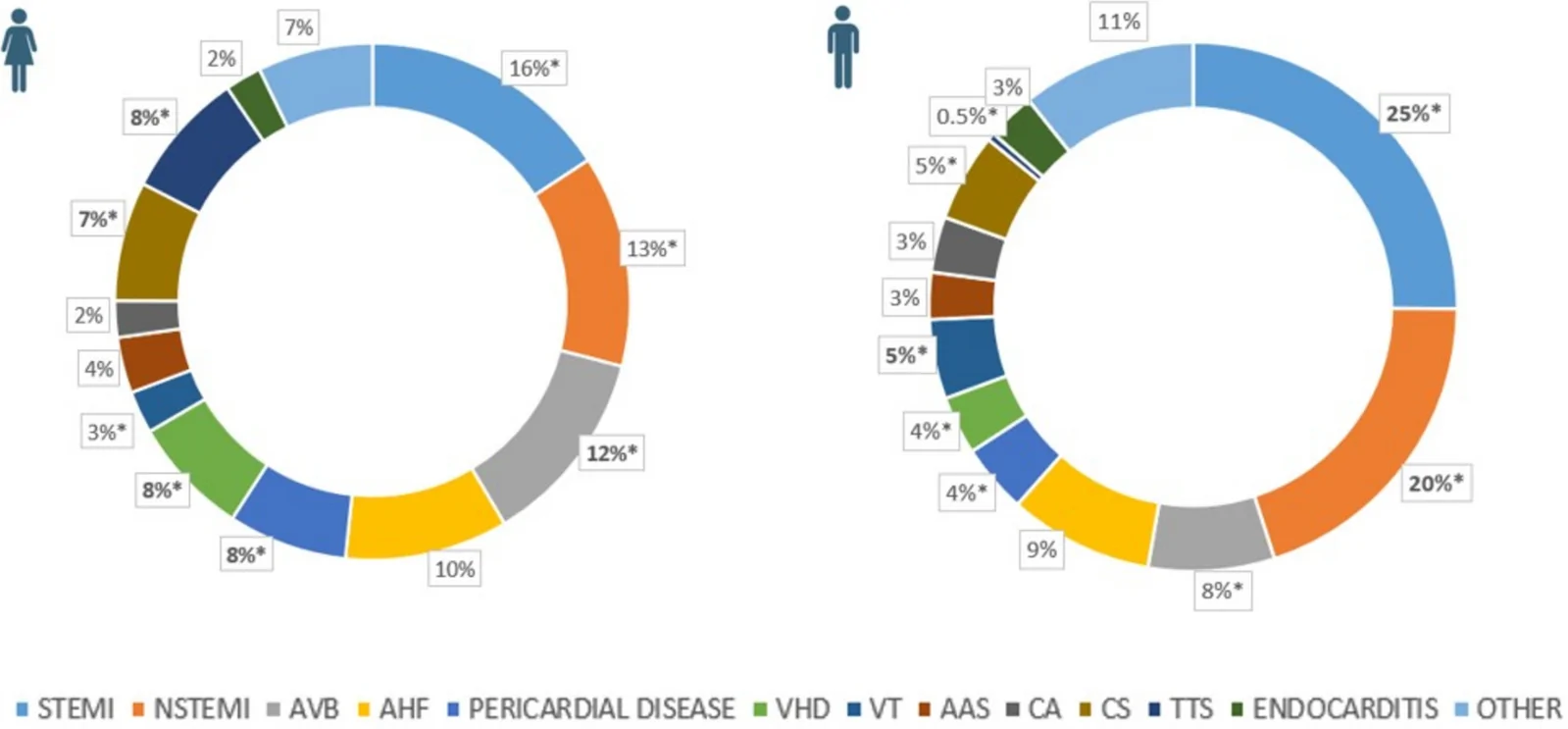
Most frequently observed acute medical conditions requiring CICU hospitalization (%) according to sex (left females, right males). Bold labels indicate conditions where gender differences are most pronounced in the respective group. An asterisk (*) denotes statistically significant differences between males and females

Conversely, women were less likely than men to be diagnosed with STEMI (16.1% vs. 26.6%, *p* < 0.0001), NSTEMI (15.3% vs. 21.7%, *p* < 0.0001), unstable angina (UA, 1.2% vs. 3.3%, *p* = 0.001), infective endocarditis (3.2% vs. 5.7%, *p* = 0.006), ventricular tachycardia (VT, 3% vs. 6.1%, *p* = 0.001), and pacemaker/implantable cardioverter-defibrillator (PMK/ICD) complications (3.8% vs. 7.8%, *p* < 0.0001). These results are detailed in Table 3 and illustrated in Fig. 1.

**Table 3:** Most frequently observed acute medical conditions requiring CICU hospitalization

Primary Reason for Admission	Female (n)	Female (%)	Male (n)	Male (%)	Total (n)	Total (%)	Fisher *p*-value
STEMI	**131**	**15.8%**	**429**	**25.1%**	**560**	**22%**	** < 0.0001**
NSTEMI	**110**	**13.2%**	**340**	**20%**	**450**	**17.9%**	** < 0.0001**
AVB	**103**	**12.4%**	**132**	**7.7%**	**235**	**9.2%**	** < 0.0001**
AHF	85	10.2%	151	8.8%	236	9.28%	0.274
PERICARDIAL DISEASE	**62**	**7.5%**	**72**	**4.2%**	**134**	**5.27%**	**0.001**
AVHD	**62**	**7.5%**	**61**	**3.6%**	**123**	**4.84%**	** < 0.0001**
VT	**22**	**2.6%**	**83**	**4.8%**	**105**	**4.13%**	**0.008**
AAS	29	3.5%	49	2.9%	78	3%	0.393
CA	19	2.3%	58	3.4%	77	3%	0.140
CS	**62**	**7.46**	**92**	**5.38**	**154**	**6%**	**0.042**
TTS	**66**	**7.9%**	**8**	**0.5%**	**74**	**2.91%**	** < 0.0001**
ENDOCARDITIS	19	2.3%	50	2.9%	69	2.7%	0.435
EXTRACTION	**9**	**1.1%**	**56**	**3.3%**	**65**	**2.55%**	**0.001**
UA	**8**	**0.9%**	**46**	**2.7%**	**54**	**2.1%**	**0.005**
MYOCARDITIS	12	1.4%	41	2.4%	53	2%	0.139
PE	**14**	**1.7%**	**13**	**0.8%**	**27**	**1.06%**	**0.039**
SEPTIC SHOCK	4	0.5%	14	0.8%	18	0.7%	0.453
MECH. COMPL. AMI	6	0.7%	10	0.6%	16	0.63%	0.790
CARDIAC MASSES	4	0.5%	2	0.1%	6	0.2%	0.094
SCAD	3	0.4%	1	0.1%	4	0.15%	0.105

*AAS* acute aortic syndrome, *AHF* acute heart failure, *AVB* atrioventricular block, *CA* cardiac arrest, *CS* cardiogenic shock, *EXTRACTION *PM/ICD lead percutaneous extraction, *MECH. COMPL. AMI* mechanical complication of myocardial infarction (interventricular septum rupture, papillary muscle rupture, free wall rupture), *PE* pulmonary embolism, *PERICARDIAL DISEASE* pericardial effusion/tamponade, pericarditis, *NSTEMI* non-ST-elevation myocardial infarction, *SCAD* spontaneous coronary artery dissection, *STEMI* ST-elevation myocardial infarction, *TTS* Takotsubo syndrome, *UA* unstable angina, *AVHD* acute presentations of valvular heart disease, *VT* ventricular tachycardia

Several non-cardiac conditions which contributed to CICU stay were more represented in women including autoimmune diseases (2.9% vs. 1.3%, *p* = 0.011)), chronic obstructive pulmonary disease (COPD, 6% vs. 3.7%, *p* = 0.011), malignancy (9.6% vs. 6.9%, *p* = 0.018), pericardial disease (8.6% vs. 4.7%, *p*-value < 0.0001), PE (3.9% vs. 1.6%, *p*-value < 0.0001), and AVHD (22% vs. 12.9%, *p*-value < 0.0001). Conversely, compared to men, women were less likely to present with other medical conditions, i.e., chronic kidney disease (CKD 8.5% vs. 11.3%, *p*-value = 0.037) and type II DM (24.1% vs. 30.5%, *p* < 0.001). These results are detailed in Table 4.

**Table 4:** Other conditions contributing to hospitalization according to sex

Condition (ICD9 Diagnosis)	Female (n)	Female (%)	Male (n)	Male (%)	Total (n)	Total (%)	Fisher *P*-value
DM	**176**	**24.1**	**466**	**30.5**	**642**	**25.2%**	** < 0.001**
AF	148	17.8	257	15.02	405	15.9%	0.074
CKD	**71**	**8.54**	**193**	**11.28**	**264**	**10%**	**0.037**
CANCER	**80**	**9.62**	**118**	**6.9**	**198**	**7.8%**	**0.018**
OBESITY	58	6.97	100	5.84	158	6.2%	0.293
PERICARDIAL DISEASE	**72**	**8.66**	**81**	**4.73**	**153**	**6%**	** < 0.0001**
ENDOCARDITIS	**27**	**3.24**	**98**	**5.73**	**125**	**4.91%**	**0.006**
COPD	**50**	**6.01**	**64**	**3.74**	**114**	**4.48%**	**0.011**
SEPTIC SHOCK	19	2.28	59	3.45	78	3%	0.141
AAS	28	3.36	39	2.28	67	2.63%	0.114
PE	**33**	**3.97**	**28**	**1.63**	**61**	**2.4%**	** < 0.0001**
AD	**24**	**2.88**	**23**	**1.34**	**47**	**1.85%**	**0.011**
PH	**4**	**0.48**	**1**	**0.058**	**5**	**0.19%**	**0.042**

*AAS*  acute aortic syndrome, *AD* autoimmune disease, *AHF* acute heart failure, *AF* atrial fibrillation, *AVB* atrioventricular block, *CA* cardiac arrest, *CCS* chronic coronary syndromes, *CKD* chronic kidney disease, *CMP* cardiomyopathies, *COPD* chronic obstructive pulmonary disease, *CS* cardiogenic shock, *DM* diabetes mellitus, *EXTRACTION* PM/ICD lead percutaneous extraction, *MECH. COMPL. AMI* mechanical complication of myocardial infarction (interventricular septum rupture, papillary muscle rupture, free wall rupture), *PE* pulmonary embolism, *PERICARDIAL DISEASE* pericardial effusion/tamponade, pericarditis, *PH* pulmonary hypertension, *NSTEMI* Non-ST-elevation myocardial infarction, *SCAD* spontaneous coronary artery dissection, *STEMI* ST-elevation myocardial infarction, *TTS* takotsubo syndrome, *UA* unstable angina, *AVHD* acute presentations of valvular heart disease, *VT* ventricular tachycardia

### Procedures performed

The most frequent procedures performed among women were coronary angiography (*n* = 114 for STEMI diagnosis, *n* = 96 for NSTEMI diagnosis) and percutaneous coronary intervention (PCI, *n* = 102 for STEMI diagnosis, *n* = 63 for NSTEMI diagnosis), PMK insertion (*n* = 81 for AVB diagnosis), valvular repair/replacement (*n* = 85 for AVHD diagnosis) and pericardiocentesis (*n* = 52). Compared to men, women with a diagnosis of STEMI were less likely to undergo coronary angiography (91.6% vs. 85%, *p*-value 0.032), PCI (86.8% vs. 76.1%, *p*-value 0.004), and Coronary Artery Bypass Grafting (CABG, 9.7% vs. 3.9%, *p*-value 0.041). The use of advanced mechanical support (MCS), including intra-aortic balloon pump (IABP), Impella, and other modalities such as extracorporeal membrane oxygenation (ECMO), showed no significant differences between the two groups across all clinical settings examined. These results are condensed in Table 5.

**Table 5:** Procedures performed according to sex

Condition (ICD9 Diagnosis)	Procedure	Female (n procedures/total ICD9)	M (n procedures/total ICD9)	Fisher *p*-value	Odds ratio (CI 95%)
AAS	Aortic surgery	2/28	2/39	1	1.423
AHF	CRRT	9/373	21/862	1	0.990
	IABP	13/373	40/862	0.445	0.742
	IMPELLA	8/373	17/862	0.828	1.089
	MV	13/373	48/862	0.152	0.612
	NIMV	8/373	7/862	0.084	2.677
	PAC	2/373	3/862	0.641	1.544
	BV	2/373	5/862	1	0.924
AVB	PM	81/122	102/154	1	1.007
CA	ICA	14/27	47/74	0.359	0.619
	MV	4/27	25/74	0.082	0.341
	CRRT	9/27	21/74	1	0.990
	IABP	13/27	40/74	0.445	0.742
	IMPELLA	8/27	17/74	0.828	1.089
	MV	13/27	48/74	0.152	0.612
	NIMV	8/27	7/74	0.084	2.677
	PAC	2/27	3/74	0.641	1.544
	BV	2/27	5/74	1	0.924
CCS	ICA	35/69	162/332	0.793	1.080
	CABG	1/69	6/332	1	0.799
	PCI	23/69	107/332	0.888	1.051
CKD	CRRT	11/71	27/193	0.843	1.127
CS	IABP	7/62	12/92	0.807	0.848
	IMPELLA	6/62	15/92	0.339	0.550
	VM	8/62	17/92	0.384	0.654
	NIMV	1/62	2/92	1	0.738
	PAC	1/62	1/92	1	1.492
	BV	2/62	2/92	1	1.5
NSTEMI	**CABG**	**5/127**	**36/371**	**0.041**	**0.381**
	ICA	96/127	287/371	0.715	0.906
	IABP	5/127	23/371	0.503	0.620
	IMPELLA	3/127	9/371	1	0.973
	PCI	63/127	163/371	0.302	1.256
PT	PP	52/72	51/81	0.233	1.529
STEMI	**PCI**	**102/134**	**395/455**	**0.004**	**0.484**
	**ICA**	**114/134**	**417/455**	**0.032**	**0.519**
	IMPELLA	1/134	6/455	1	0.563
	IABP	7/134	16/455	0.445	1.512
	MV	4/134	17/455	0.797	0.793
AVHD	AVR	59/183	52/220	0.058	1.537
	MVR	16/183	26/220	0.331	0.715
	VPL	8/183	7/220	0.602	1.391
VT	TCA	2/25	16/105	0.523	0.484

*AAS* acute aortic syndrome, *AHF* acute heart failure, *AVB* atrioventricular block, *AVR* aortic valve replacement, *BV* balloon valvuloplasty, *CA* cardiac arrest, *CCS* chronic coronary syndromes, *CKD* chronic kidney disease, *CRRT* continuous renal replacement therapy, *CS* cardiogenic shock, *IABP* intra-aortic balloon counterpulsation, *ICA* invasive coronary angiography, *MV* mechanical ventilation, *MVR* mitral valve replacement, *NIMV* non-invasive mechanical ventilation, *PAC* pulmonary artery catheter, *PE* pulmonary embolism, *PP* percutaneous pericardiocentesis, *PT* pericardial tamponade, *NSTEMI* Non-ST-elevation myocardial infarction, *STEMI* ST-elevation myocardial infarction, *TCA* transcatheter ablation, *AVHD* acute presentations of valvular heart disease, *VPL* valvuloplasty, *VT* ventricular tachycardia

### Length of hospital stay

The average length of stay was 5.2 days for females and 5.6 days for males. This difference was not statistically significant (*p* = 0.18), as shown in Table 6.

**Table 6:** Length of stays in CICU related to sex

Sex	N	LOS (Days, mean)	SD	*P*-Value
Male	1710	5.632	8.165	0.180
Female	831	5.191	6.879	

*LOS* length of stay

### Mortality rates in the CICU

In-CICU mortality was similar in females and men (7.9% in females vs. 5.9% in males, *p* = n.s.). However, females had a significantly higher in-CICU mortality rate for STEMI (11.9% vs. 4.2%, *p* = 0.0003) and PE (27.3% vs. 3.6%, *p* = 0.016). A summary of these findings is provided in Tables 7 and 8. The most frequent diagnoses in female patients dying in CICU were AHF (63.9%), CS (43.2%), STEMI (26.2%), and AVHD (26.2%), as shown in Table 9.

**Table 7:** Mortality rate in CICU related to sex

Sex	N_tot_	Deaths (n, %)	Pearson Chi-Square	*P*-value	Odds ratio (CI 95%)
Male	1710	102 (5.96)	0.184	0.196	1.541
Female	831	61 (7.34)

**Table 8:** CICU mortality for specific conditions according to sex

Condition (ICD9 diagnosis)	Female (n/cases)	M (n/cases)	Fisher *p*-value	Odds ratio (CI 95%)
AAS	3/28	3/39	0.688	1.244
AHF	39/373	68/862	0.15	1.3
AVB	4/122	9/154	0.398	1.271
CA	11/27	18/74	0.137	1.31
CS	30/62	34/92	0.184	1.119
ENDOCARDITIS	7/19	8/50	0.19	1.279
MECH. COMPL. AMI	1/7	1/12	1	1.248
NSTEMI	10/127	18/371	0.265	1.236
PE	**9/33**	**1/28**	**0.016**	**1.207**
PERICARDIAL DISEASE	4/72	8/81	0.379	1.236
SEPTIC SHOCK	8/19	19/59	0.58	1.339
STEMI	**16/134**	**19/455**	**0.003**	**1.233**
TTS	3/67	1/10	0.434	1.276
AVHD	16/183	20/220	1	1.196
VT	2/25	7/105	0.683	1.254

*AAS* acute aortic syndrome, *AHF* acute heart failure, *AVB* atrioventricular block, *CA* cardiac arrest, *CS* cardiogenic shock, *MECH. COMPL. AMI* mechanical complication of myocardial infarction (interventricular septum rupture, papillary muscle rupture, free wall rupture), *PE* pulmonary embolism, *PERICARDIAL DISEASE *pericardial effusion/tamponade, pericarditis, *NSTEMI* non-ST-elevation myocardial infarction, *STEMI* ST-elevation myocardial infarction, *TTS* Takotsubo syndromem, *AVHD* acute presentations of valvular heart disease, *VT* ventricular tachycardia

**Table 9:** CICU mortality for diverse conditions in the female population (descending order)

ICD9 Diagnosis	F, n	% of Tot Female Deaths
AHF	39	63.9
CS	30	49.2
AF	16	26.2
STEMI	16	26.2
AVHD	16	26.2
PE	9	14.8
DM	8	13.1
SEPTIC SHOCK	8	13.1
CCS	7	11.5
ENDOCARD	7	11.5
NSTEMI	10	16.4
INFECTIOUS	10	16.4
CKD	11	18
CA	11	18
AVB	4	6.6
COPD	4	6.6
CANCER	4	6.6
PERICARDIAL DISEASE	4	6.6
AAS	3	4.9
DEVICE	3	4.9
STROKE	3	4.9
TTS	3	4.9
MEC_COM_AMI	1	1.6
AMYLOIDOSIS	2	3.3
TV	2	3.3

*AAS* acute aortic syndrome, *AHF* acute heart failure, *AVB* atrioventricular block, *CA* cardiac arrest, *CS* cardiogenic shock, *MECH. COMPL. AMI* mechanical complication of myocardial infarction (interventricular septum rupture, papillary muscle rupture, free wall rupture), *PE* pulmonary embolism, *PERICARDIAL DISEASE* pericardial effusion/tamponade, pericarditis, *NSTEMI* non-ST-elevation myocardial infarction, *STEMI* ST-elevation myocardial infarction, *TTS* Takotsubo syndrome, *AVHD* acute presentations of valvular heart disease, *VT* ventricular tachycardia

### Long-term mortality

**Fig. 2:**
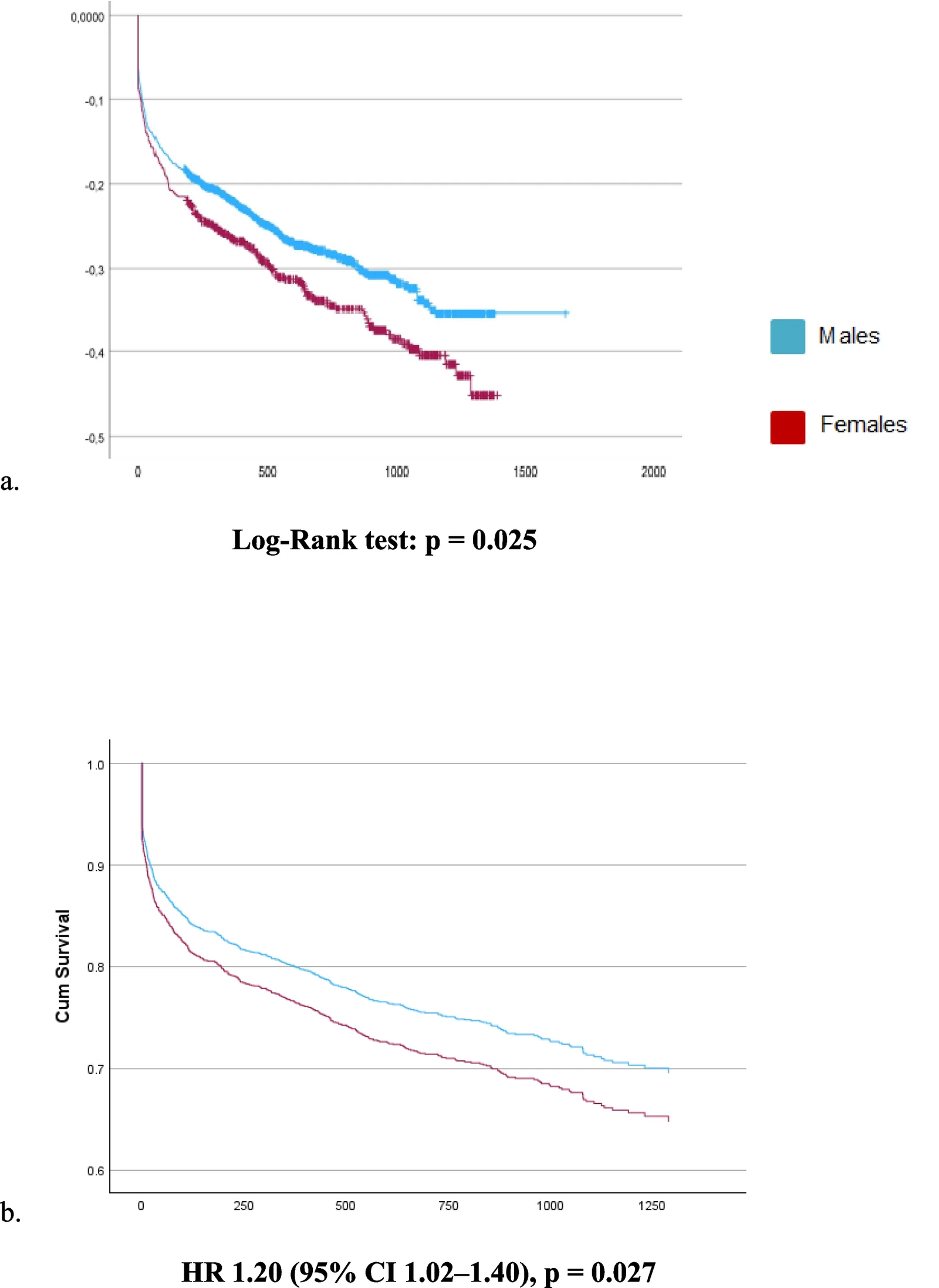
**a**)Kaplan–Meier survival curves according to sex.Survival significantly differed between groups (Log-Rank *p* = 0.025).**b**)In the univariable Cox regression analysis, female sex was associated with a higher risk (HR 1.20, 95% CI 1.02–1.40; *p* = 0.027)

The analysis of long-term survival following admission to the CICU during the follow-up period from November 2020 to October 2024 revealed a significantly higher long-term mortality rate in women (Log-Rank test: *p* = 0.025). In the univariable Cox proportional hazards model, female sex was associated with an increased risk (HR 1.20, 95% CI 1.02–1.40; *p* = 0.027), as shown in Fig. 2. The mean survival time was notably shorter in women (1,010 days; 95% CI: 970–1,050) compared to men (1,250 days; 95% CI: 1,216–1,284). Disease-specific long-term survival revealed no significant difference between males and females (Supplementary Material 3).

## Discussion

In our study, we provide detailed, sex-specific insights into acute cardiac care needs enhancing our understanding of current trajectories of female patients in critical cardiac care. We observed notable sex-based differences among patients admitted to the CICU. The lower representation of women in the CICU, accounting for only one-third of admissions, may be explained by several interrelated factors. One potential reason is the delayed onset and atypical presentation of cardiovascular disease in women, which may lead to delayed recognition, underdiagnosis, or misclassification of their condition, ultimately reducing the likelihood of CICU admission [2]. Another contributing factor may be differences in referral patterns and treatment approaches. There is evidence suggesting that women with acute coronary syndromes or other critical cardiac conditions are less likely to be referred for early aggressive interventions [3]. This disparity may be driven by both patient-related factors, such as delays in seeking medical attention, and physician-related factors, including the underestimation of cardiovascular risk in women. Furthermore, sex-based differences in the prevalence and severity of critical cardiac conditions may also play a role. Women are less frequently affected by acute coronary syndromes, which are among the most common reasons for CICU admission. Instead, they are more likely to present conditions which, although serious, may not always necessitate intensive care [4]. Sociocultural and behavioural factors may further influence these differences. Women are often more likely to delay seeking medical care due to caregiving responsibilities, lower perceived risk of cardiovascular disease, or differences in health-seeking behaviours. These delays could lead to more advanced but subacute disease presentations, potentially resulting in alternative hospitalization pathways rather than direct admission to intensive care [5]. Moreover, triage protocols and risk scores, historically developed based on predominantly male populations, may underpredict the need for intensive care in women, leading to potential misallocation of hospital resources.

Women admitted to CICU were significantly older than men, a finding consistent with previous literature, indicating that women typically present with cardiovascular conditions at an older age than men. This age disparity may be attributed to the protective effects of oestrogen before menopause and the delayed onset of cardiovascular disease in women [6].

Our results highlight significant sex-related variations regarding cardiovascular risk factors. Women were less likely to be active or former smokers compared to men, a trend that aligns with broader epidemiological studies suggesting lower smoking rates among women [4]. This difference may be partially explained by historical and sociocultural factors influencing smoking behaviours in men and women. Conversely, obesity and DM were more prevalent in women than in men, reinforcing existing evidence that suggests sex-related differences in body composition, and metabolic risk factors [7]. Despite these differences, no significant sex-based variations were observed in the prevalence of other traditional cardiovascular risk factors, including hypertension, dyslipidemia, and family history of heart disease, suggesting that these factors play a similarly important role in both men and women admitted to the CICU. The observed differences in traditional cardiovascular risk factors between men and women suggest that sex-specific prevention and intervention strategies may be warranted to optimize patient outcomes.

Our study highlights significant sex-based differences in the clinical presentation of acute cardiac conditions requiring hospitalization. In absolute terms, the most frequent acute cardiac conditions in female patients were AHF, AVHD, AF, STEMI, and NSTEMI. However, in relative terms, women were more likely than men to present with AVB, TTS, pericardial effusion or tamponade, acute presentation of valvular disease, PE and CS. These findings reinforce the distinct pathophysiology and clinical trajectories of cardiovascular disease in women.

The predominance of AHF and AVHD among female patients is in line with prior research, which suggests that women are more frequently affected by heart failure with preserved ejection fraction and degenerative valvular disease, particularly mitral and aortic stenosis [8]. These differences may be attributed to age-related structural cardiac changes, hypertension, and obesity, which contribute to diastolic dysfunction. Similarly, the higher prevalence of AF in women could be explained by their older age at presentation, greater susceptibility to atrial remodeling, and higher burden of hypertension.

Our data also confirm the higher occurrence of conduction abnormalities, stress cardiomyopathy, degenerative valvular diseases, and thromboembolism in female patients, likely reflecting their older age, greater vulnerability to stress-related conditions, and the hormonal effects of estrogen on the cardiovascular system. The increased relative prevalence of AVB in women may be explained by sex-based differences in electrophysiology and a greater predisposition to degenerative fibrosis [9]. The strong association between TTS and female sex is well-documented, with women representing the vast majority of cases [10]. Although the exact mechanisms remain unclear, proposed hypotheses include heightened sympathetic activation, hormonal influences, and endothelial dysfunction, all of which may contribute to the pathophysiology of this stress-related cardiomyopathy. Similarly, the increased occurrence of pericardial effusion or tamponade in women may be linked to their greater susceptibility to oncological or autoimmune disorders, such as systemic lupus erythematosus and rheumatoid arthritis, which frequently involve pericardial involvement. In our study, both conditions demonstrated a higher prevalence in females. Hormonal factors, including oral contraceptive and hormone replacement therapy use, pregnancy-related hypercoagulability, and sex-related differences in venous thromboembolism risk [11], could influence the higher relative prevalence of PE in female patients.

A particularly noteworthy finding is the higher incidence of CS in female patients despite their lower prevalence of acute coronary syndromes, which is a frequent determinant of CS. This paradox aligns with prior evidence indicating that women with STEMI have higher rates of AHF, left ventricular dysfunction, and CS, even after adjusting for other clinical factors [12]. Several mechanisms may contribute to this increased risk, including delayed diagnosis, prolonged ischemic time, and a higher burden of endothelial dysfunction and microvascular disease, which may predispose women to hemodynamic instability. Women with myocardial infarction also frequently experience atypical symptoms, such as fatigue, abdominal discomfort, nausea, and dizziness, which may lead to reduced awareness, delays in seeking medical attention, and an increased likelihood of prehospital CS [13]. Additionally, the later onset of coronary artery disease in women, combined with their greater susceptibility to microvascular dysfunction, may further contribute to MI complications, including CS.

On the other hand, female patients were less likely to be admitted for acute coronary syndromes and ventricular arrhythmias. These data confirm previous literature on female protection against coronary artery disease, historically considered a male-predominant disease. The lower incidence of ventricular arrhythmias in women is well-established in modern literature, especially in the setting of post-acute myocardial infarction [14]. The underlying reasons for sex differences in the occurrence of ventricular arrhythmias have not been fully identified. Still, several studies have shown a higher incidence of previous myocardial infarction and ischemic heart disease in men, potentially leading to a larger myocardial scar burden that serves as a substrate for re-entrant circuits [15].

The present study highlights significant differences in the management of acute cardiovascular conditions between men and women, particularly in the utilization of invasive procedures. Among women, the most frequently performed interventions included coronary angiography and PCI for both STEMI and NSTEMI, PMK insertion for AVB, valvular repair/replacement for valvular heart disease, and pericardiocentesis for pericardial effusion.

Notably, when compared to men, women diagnosed with STEMI were significantly less likely to undergo coronary angiography, PCI, and CABG. The lower rates of these procedures among women (85% vs. 91.6% for coronary angiography, 76.1% vs. 86.8% for PCI, and 3.93% vs. 9.7% for CABG) suggest potential disparities in treatment strategies. These differences could be attributed to multiple factors, including biological sex-related variations in coronary artery disease presentation, underestimation of cardiovascular risk in women, and potential sex bias in clinical decision-making [16].

Our study reveals significant divergencies in the types and frequencies of procedures performed in female patients compared to the male cohort, indicating differences in both clinical presentation and therapeutic approaches. Our results highlight that women are less likely to undergo procedures commonly associated with STEMI, such as coronary angiography and PCI, and surgical coronary revascularization, i.e., CABG, in the setting of NSTEMI. Our results align with recent literature about sex differences in acute cardiovascular care. Women with acute myocardial infarction were less likely to receive reperfusion and revascularization and less frequently received guideline-recommended pharmacological therapies at discharge [17].

As already mentioned, women with acute myocardial infarction are more likely to experience delayed presentation (often beyond the 48-h window for revascularization) and have a higher burden of comorbidities, which may partly explain the less aggressive revascularization strategy observed in this cohort. Additionally, the higher incidence of myocardial infarction with non-obstructive coronary arteries, spontaneous coronary artery dissection, vasospastic angina, and microvascular dysfunction in women may contribute to the reduced use of revascularization procedures. As far as CABG is concerned, evidence proved that females are less likely to receive multiple arterial grafts and complete revascularization compared to men during CABG surgery [18]. The limited use of CABG in our female cohort may be ascribed to the lower incidence of multivessel disease. Furthermore, the older age of this group (besides representing a potential confounding factor) may influence both patient and physician preferences for less invasive interventions, often due to increased perioperative risk and longer recovery times associated with surgical interventions.

Interestingly, in our institution, the use of MCS disclosed no significant differences between the two groups under examination in any setting (AHF, CS, ACS). The limited number of MCS device implantations may have constrained the robustness of our findings. Nowadays, data regarding sex differences in MCS are controversial. In a recent study evaluating sex differences in MCS in acute myocardial infarction-related CS via the National Inpatient Sample database, there was no sex disparity in the use of MCS or the timing of placement, even after adjusting for baseline and hospital characteristics [19]. On the other hand, a 2024 meta-analysis from Fisher et al. proved that female patients with cardiogenic shock, both from acute myocardial infarction and AHF, were 30% less likely to receive MCS [20].

Our analysis of the length of hospital stay in female patients admitted to CICU does not reveal significant sex-related differences, confirming data from other studies [21]. Interestingly, our findings showed that long-term mortality, assessed over a follow-up period spanning from CICU admission to a maximum of approximately 4 years, was significantly higher in women compared to men. Nonetheless, we found no significant differences in overall crude mortality between male and female patients during the index hospitalization, but only a non-significant trend toward higher in-hospital mortality in women, which may reflect a limitation of our study, such as sample size constraints or residual confounding factors. Female patients exhibited significantly higher mortality rates compared to their male counterparts when admitted with a diagnosis of PE. As mentioned, variations in the prothrombotic state because of a diverse hormonal milieu may influence female vulnerability and complications of thromboembolism, determining a worse outcome in this specific setting. Equally, our female cohort showed substantially increased mortality rates than male patients when hospitalized for STEMI. Recent evidence discloses that there exists a significant sex disparity in myocardial infarction outcomes [5]. This disparity might be attributed to several factors, including the fact that women often present with STEMI at an older age and with more comorbidities, such as hypertension and heart failure, which may complicate their clinical management. Additionally, women are more likely to present with unusual symptoms, leading to delays in diagnosis and treatment, resulting in longer ischemic times and missed opportunities for timely reperfusion therapy. Finally, women tend to receive less aggressive treatment strategies, including lower rates of coronary angiography, revascularization procedures, and guideline-recommended pharmacological therapies, which could further contribute to the worse outcomes observed in this population [3]. Nevertheless, even after adjusting for demographic factors and cardiovascular risk profiles, women still exhibited higher mortality rates, implying that fundamental physiological differences between males and females are not fully accounted for in current diagnostic and treatment guidelines [13]. Our findings suggest that sex-related differences in outcomes among CICU patients may become more evident over time rather than during the acute phase of hospitalization. The comparable in-hospital crude mortality rates between men and women, despite the trend toward higher mortality in females, may indicate that short-term outcomes are influenced by factors beyond sex alone, such as acute management strategies or baseline clinical characteristics. Conversely, the significantly higher long-term mortality observed in women raises important questions about potential disparities in post-discharge care, differential disease progression, or underlying biological differences. As mentioned, women are older than their counterparts and this aspect may explain an excess of mortality both during CICU hospitalization and in the post-discharge phase.

Our diagnosis-specific outcome analyses indicate that the majority of deaths in absolute terms among female patients in the CICU occur in those diagnosed with AHF, CS, STEMI, and AVHD. This epidemiological data could assist the targeted prioritisation of healthcare resources to optimise female patient outcomes and resource allocation. For instance, optimising reperfusion strategies in STEMI, implementing sex-specific risk stratification protocols to ensure timely access to advanced hemodynamic support in cases of CS, and more aggressive management of valvular disease before it progresses to an acute state could improve in-hospital prognoses among women.

### Study limitations

This study has several limitations that should be considered when interpreting the results. Firstly, the retrospective and single-centre nature of the research introduces potential biases and restricts the broader applicability of the findings. Since the study was conducted at a single institution, our results may reflect local practices that may not be fully representative of other healthcare settings. Moreover, using ICD-9 codes for diagnosing and categorizing conditions has inherent drawbacks, as the accuracy and thoroughness of coding can vary. This could lead to under-reporting or misclassification of diagnoses, particularly in cases involving complex, multimorbid conditions. Finally, our study aimed to explore the health needs of the female population from an epidemiological perspective. As a descriptive, hypothesis-generating study, all comparisons are unadjusted, and observed sex-related differences may partly reflect variations in age or other unmeasured factors. Consequently, our findings provide valuable insights into sex-specific differences in CICU admissions and outcomes, but they do not allow for conclusions regarding the underlying mechanisms driving these disparities. This limitation highlights the need for future prospective and mechanistic studies to better understand the factors contributing to the observed differences.

## Conclusions

Our study highlights significant sex-specific differences in patients admitted to the CICU. Women constituted only one-third of the total cohort, highlighting their marked underrepresentation compared to men. By assessing the full spectrum of cardiovascular conditions rather than focusing on a single disease, our study was able to comprehensively capture this important aspect of sex-related disparities in CICU admissions. Women were older, had a distinct cardiovascular risk profile, and exhibited different primary reasons for admission compared to men. They were more frequently admitted for AVB, TTS, AVHD, PE, and pericardial diseases, whereas men were more often admitted for acute coronary syndromes and ventricular arrhythmias. Procedural disparities were evident, with women being less likely to undergo coronary angiography, PCI, and CABG, particularly in STEMI and NSTEMI. While overall in-CICU mortality was similar, women had significantly higher mortality rates for STEMI and PE. Furthermore, our diagnosis-specific outcome analyses reveal that the majority of deaths among female patients occurred in those diagnosed with AHF, CS, STEMI, and AVHD, with CS and AVHD more frequently observed in women upon CICU admission compared to men. Long-term survival analysis further demonstrated increased mortality in women, emphasizing the critical need to investigate sex-specific differences in timely diagnosis, admission patterns, therapeutic interventions, and post-discharge care. Collectively, our findings offer a comprehensive perspective on acute cardiac care in women, offering epidemiological insights that may inform targeted strategies to enhance management and improve outcomes for female patients in the CICU.

## Supplementary Information

Below is the link to the electronic supplementary material.

ESM 1 (DOCX 543 KB)

## Data Availability

The data underlying this article will be shared on reasonable request to the corresponding author.
