# Supplementary material for: Sex-related presentation, outcome, and treatment in CICU: the SPOTlight STUDY
Source: Clin Res Cardiol. 2025 Dec 8;115(11):1930–43. doi: 10.1007/s00392-025-02816-z (PMC13658359; doi:10.1007/s00392-025-02816-z)
Supplement: Supplementary file 1 [file 392_2025_2816_MOESM1_ESM.docx]

**SUPPLEMENTARY MATERIAL**

**1. Admission criteria for CICU:**

a) ***Therapies and Devices Requirements***:

- Need for high-concentration oxygen (>50%).
- Use of inotropes or intravenous vasopressors/antihypertensives.
- Invasive arterial or central venous pressure monitoring.
- Mechanical ventilation (invasive or non-invasive).
- Mechanical circulatory support.
- Continuous renal replacement therapy (CRRT).
- Temporary transvenous or transcutaneous pacing.
- Post-cardioversion for unstable ventricular arrhythmias.
- Swan-Ganz catheter placement.
- Temperature control systems (targeted temperature management).

***b) Condition-Specific Risks***:

- STEMI:

1. Intermediate to high-risk post-primary PCI.
2. Systemic thrombolysis awaiting invasive approach.
3. Severe heart failure or late presentation.

- NSTEMI:

1. Dynamic ST elevation in aVR or Wellens’ pattern.
2. Persistent angina, arrhythmias, or severe heart failure.
3. Heart Failure and Shock.

- Cardiogenic shock (INTERMACS 1–3).
- Distributive, obstructive, or mixed shock.
- Valvular Heart Disease:

1. Severe aortic stenosis with syncope.
2. Severe regurgitant or obstructive valvular lesions with moderate to severe heart failure.

- Atrial Arrhythmias:

1. Need for intravenous rate-control drugs.
2. Hemodynamic instability or severe heart failure.

- Ventricular Arrhythmias:

1. Symptomatic non-sustained ventricular tachycardia.
2. Sustained ventricular tachycardia without cardiac arrest.

- Atrioventricular Conduction Disorders:

1. Complete heart block with wide QRS rhythm.
2. Unstable third-degree AV block.

- Pericardial Diseases:

1. Clinical or echocardiographic signs of tamponade.
2. Severe volume overload in pericardial constriction.

- Endocarditis:

1. Severe valvular insufficiency or obstruction.
2. Systemic embolization or aortic valve abscess.

- Aortic Dissection and Hypertensive Crises:

1. Acute type B dissection or chronic dissection with new symptoms.
2. Hypertensive crises requiring close monitoring.
3. Acute type A dissection undergoing medical management

***c) Post-Procedural Monitoring***:

- Complications from coronary angioplasty or electrophysiology procedures.
- Percutaneous valve repair or replacement.
- Structural or congenital interventional cardiology procedures.

2. **STROBE-compliant flow diagram illustrating the selection of the study population.**

**3. Gemelli GENERATOR Real World Data (G2 RWD) Facility**

The Gemelli GENERATOR Real World Data (G2 RWD) Facility is responsible for the design and execution of the data-driven study, together with the PI and the clinical research team. A standardized approach to setting up a patient-centred data mart using retrospectively collected information will be adopted. Risk profiles will be defined by integrating patient comorbidities and risk factors with a multimodal view, using a standardized stepwise methodology defined at G2 RWD facility:

•	execution of automated procedures for data extraction from hospital operational systems and warehouse. Any such processing is performed with anonymized data for what concerns Generator RWD staff activities;

•	quality and semantic consistency check;

•	data transformation (e.g., from text to categorical as is the case when transforming medical reports into actionable data through text mining) and integration in the target data mart model;

•	analytics procedures to highlight first-round real-world evidence, identify correlations, and provide descriptive analysis to the clinicians;

•	A.I. model development including statistical and machine learning, clustering techniques, and neural networks. A schematic view of the Generator Real World Data standard framework is shown below.

*Ethical And Privacy Standards*

The study has been executed using Generator Real World Data standard methodologies including successful completion of Data Protection Impact Analysis (DPIA), performed in May 2021. Therefore, the study is compliant with GDPR Italian and European directives and regulations (EU Directive 2016/679 and under Italian Laws: Decreto Legislativo 196/2003 and its amendment Decreto Legislativo 101 2018). The DPIA allows the treatment of data on health for research purposes also without the consent of the patient.

The guidelines for data privacy are covered by the following documents:

•	Generator main document “Linee Guida Utilizzo Real World Data” prescribes the governance mechanisms for Generator through two main bodies (Comitato di Supervisione and Comitato Operativo) where representatives appointed from IRCSS, and other key Units of Policlinico participate and have a mandate to decide.

•	“Orizzonte Normativo Real World Data” which provides the directives for the execution of any Generator study to assure its compliance with European and Italian GDPR directives to the treatment of personal data.

•	Data Protection Impact Analysis (DPIA) for Generator Real World Data Facility as of May 31, 2021, according to art. 35 del Reg. UE 679/2016 il 31 Maggio 2021.

All RWD studies have research purposes and therefore consent-related procedures are consistent with European Directive 2016/679. The data architecture which supports RWD studies has been defined to provide the highest degree of protection, following all GDPR and security requirements. In this respect, RWD provides “protection by design” in each step of the process.

**3.Long-term survival Kaplan Meier for the primary reason for hospitalization.**

i) **AVB, atrioventricular block**

|  | Chi-Square | sig |
| --- | --- | --- |
| Log-Rank (Mantel-Cox) | 0.263 | 0.608 |
|  |  |  |

ii) **STEMI, ST-elevation myocardial infarction**

|  | Chi-Square | sig |
| --- | --- | --- |
| Log-Rank (Mantel-Cox) | 1.484 | 0.223 |
|  |  |  |

iii) **NSTEMI, non-ST-elevation myocardial infarction**

|  | Chi-Square | sig |
| --- | --- | --- |
| Log-Rank (Mantel-Cox) | 0.126 | 0.723 |
|  |  |  |

iv) **CA, cardiac arrest**

|  | Chi-Square | sig |
| --- | --- | --- |
| Log-Rank (Mantel-Cox) | 2.972 | 0.085 |
|  |  |  |

v) **CS, cardiogenic shock**

|  | Chi-Square | sig |
| --- | --- | --- |
| Log-Rank (Mantel-Cox) | 1.283 | 0.257 |
|  |  |  |

vi) **AHF, acute heart failure**

|  | Chi-Square | sig |
| --- | --- | --- |
| Log-Rank (Mantel-Cox) | 1.023 | 0.312 |
|  |  |  |

vii) **TTS, Takotsubo syndrome**

|  | Chi-Square | sig |
| --- | --- | --- |
| Log-Rank (Mantel-Cox) | 0.12 | 0.912 |
|  |  |  |
